# Self-reported school experience as a predictor of self-harm during adolescence: A prospective cohort study in the South West of England (ALSPAC)

**DOI:** 10.1016/j.jad.2014.11.003

**Published:** 2015-03-01

**Authors:** Judi Kidger, Jon Heron, David A Leon, Kate Tilling, Glyn Lewis, David Gunnell

**Affiliations:** a University of Bristol, United Kingdom; b London School of Hygiene and Tropical Medicine, United Kingdom; c University College, London, United Kingdom

**Keywords:** Adolescence, Self-harm, School risk factors, ALSPAC

## Abstract

**Background:**

Several aspects of school life are thought to be associated with increased risk of self-harm in adolescence, but these have rarely been investigated in prospective studies.

**Methods:**

Members of the Avon Longitudinal Study of Parents and Children (ALSPAC) birth cohort completed postal surveys of school experiences aged 14, and self-harm behaviour aged 16 (*n*=3939). Associations between school experiences (feeling connected to school, enjoyment of school and perception of teachers as fair) and subsequent self-harm were examined using multivariable logistic regression models.

**Results:**

Self-harm aged 16 was associated with earlier perceptions of school, specifically not getting on well with or feeling accepted by others (OR=2.43 [1.76, 3.35] and OR=2.69 [2.16, 3.35] respectively), not liking school or the work done in class (OR=1.40 [1.17, 1.69] and OR=1.36 [1.10, 1.67]), and feeling that teachers are not clear about behaviour or fail to address misbehaviour consistently (OR=1.59 [1.20, 2.12] OR=1.89 [1.51, 2.37]). These associations were partially attenuated in models controlling for mental health concurrent with the outcome. Poor school experiences were related to both suicidal and non-suicidal self-harm, with slightly stronger associations visible for the former.

**Limitations:**

(i) There was some loss to follow up, (ii) experience of bullying was not measured, and (iii) exposure and outcome measures were self-report.

**Conclusions:**

Students who feel unconnected to school, unhappy at school, or feel that teachers are unfair are more likely to self-harm in the future. Assessing students׳ perceptions of school may serve to identify those at risk of self-harm who would benefit from preventative interventions.

## 1 Introduction

Self-harm during adolescence is of concern due to its strong association with psychological distress and suicidal thoughts (Kidger et al., 2012a; Landstedt and Gillander Gadin, 2011; Hawton et al., 2002), and with future self-harm and completed suicide (Hawton and Zahl, 2003). Much adolescent self-harm does not reach the attention of medical services, making community-based studies essential when examining associated risk factors. One recent study found that only 12% of 16 year olds who had reported self-harming had sought medical help following the most recent episode (Kidger et al., 2012a).

An emerging body of evidence has shown that schools can have an effect on health (Denny et al., 2011; Patton et al., 2006), including emotional health (Kidger et al., 2012b). Poor relations with peers and teachers, lack of engagement with school and school life, and poor academic achievement are all associated with increased risk of self-harm (Winsper et al., 2012; Landstedt and Gillander Gadin, 2011; Young et al., 2011; Winfree and Jiang, 2010; Wichstrom, 2009). Conversely feeling connected to or engaged with school, and feeling safe at school are associated with a decreased risk of self-harm (Fleming et al., 2007).

Most studies of school-related risk factors for self-harm have been cross sectional (Fliege et al., 2009), making it difficult to establish the temporal order of events. It may be that experiencing difficulties at school leads a young person to self-harm as a coping strategy, or that an individual who self harms increasingly finds him or herself disconnected from school life, either because of the stigma surrounding the behaviour, or because of whatever led to the behaviour in the first place. Mental disorders such as depression and anxiety are associated with difficult school experiences (Wang, 2009; Shochet et al., 2006), and with self-harm and suicidal thoughts and behaviours (Foley et al., 2006; Hawton et al., 2002, Wichstrom, 2009). Therefore one possible explanation for the link between school experiences and self-harm is that problematic experiences lead to poor mental health and poor mental health then leads an individual to self-harm. Or it could be that individuals who suffer from mental disorders such as depression and anxiety have difficulty engaging with school positively, and are also more likely to self-harm. In other words poor mental health is an underlying factor associated with both poor school experiences and self-harm.

This study uses data collected as part of a prospective longitudinal cohort study (ALSPAC) to examine the impact of school experiences on future self-harm. Although the temporal order of any associations is not able to be established due to the lack of a measure of self-harm at baseline, the study is unique in examining the association of school experiences on self-harm two years into the future. Further, it separately examines the effect of school experiences on suicide attempts and non-suicidal self-harm, whereas previous studies have not made this distinction, or have only examined one or the other.

Specifically the paper examines two questions:

1. Is there an association between experiences of school at age 14 and self-reported self-harm at age 16?
2. Does the association between school experience and subsequent self-harm differ among those who self-harm with suicidal intent, compared to those who self-harm without suicidal intent?

## 2 Methods

### 2.1 Sample

The sample comprised participants from the Avon Longitudinal Study of Parents and Children (ALSPAC: Boyd et al., 2013; Fraser et al., 2013). ALSPAC is an ongoing population-based study investigating a wide range of environmental and other influences on the health and development of children. Pregnant women resident in the former Avon Health Authority (Bristol) in South-West England, having an estimated date of delivery between 1 April 1991 and 31 December 1992 were invited to take part, resulting in a ‘core’ cohort of 13,796 singletons and first born of twins alive after one year. The study website contains details of all the data that is available through a fully searchable data dictionary (http://www.bris.ac.uk/alspac/researchers/data-access/data-dictionary/).

### 2.2 Measures

Thirty nine questions about school-related experiences were included in a self-completion postal questionnaire, sent to study participants when they were 14 years old. The questions were in the form of statements such as ‘my school is a place where I really like to go each day,’ with a choice of response boxes to tick ranging from strongly agree to strongly disagree (see Appendix A for a full list of questions).

As a large number of questions regarding school experience were asked, some of which were very similar, a factor analysis was conducted to identify a smaller group of key exposure variables that were distinct from each other, to be used in the analysis. The rotated solution with six factors was deemed adequate on the basis of fit statistics (comparative fit index [CFI]=0.962, Tucker–Lewis index [TLI]=0.949, root mean square error of approximation [RMSEA]=0.046). On further inspection, three of these factors were discarded. The reasons were as follows: one comprised only two extremely similar items referring to the appraisal of schoolwork, another consisted solely of items that asked about *feelings* in school and was therefore less informative about the school context as this could be confounded by the individual׳s general emotional state, and the third was made up of items that were a subset of those that loaded on another factor. The remaining three factors were labelled ‘connectedness to school/other students’, ‘enjoyment of school’ and ‘clear/fair boundaries’ (see Appendix B). The two items that loaded the most strongly onto each factor were included in the analysis. The use of single items rather than the whole factors enabled comparisons with previous studies that have examined the impact of similar single items on student emotional health (Kidger et al., 2012b), and helped identify specific aspects of the school experience that might be amenable to intervention.

Questions about self-harm and suicidal thoughts were included in a self-completion postal questionnaire, sent to participants when they were aged 16 years (Kidger et al., 2012a). Participants were asked “have you ever hurt yourself on purpose in any way (e.g. by taking an overdose of pills or by cutting yourself)?” Those who answered yes were asked further closed response questions regarding whether they had wanted to die the most recent time they had self-harmed, and whether they had ever seriously wanted to kill themselves while self-harming, to establish suicidal intent. No information was available on self-reported self-harm at age 14 years.

Confounders for this analysis were identified a priori based on evidence from the literature regarding risk factors for self-harm. The following variables were used:

• Gender.
• Socioeconomic position (SEP): (i) mother׳s occupational social class (manual/intermediate/non-manual); (ii) mother׳s self-reported highest educational level – A-level (post-compulsory schooling qualifications taken at age 18) or degree, O-level (examinations taken at the end of compulsory schooling around age 16 years by students deemed more academically able) or lower than O level; and (iii) household income reported by the mother when the participant was aged 11 (three categories – <£290/£290-£559/£560< per week).
• Maternal depression, measured using mother׳s score on the Edinburgh Postnatal Depression Scale (EPDS), when the participant was 11 years old (Cox et al., 1996). A cut-point of 12/13 was used as the threshold for a depressive illness (Cox et al., 1987).
• Participant׳s emotional health prior to reporting on their school-related experiences, measured using the parent completed Strengths and Difficulties Questionnaire (SDQ), emotional subscale only, administered when aged 13. A cut off of 4/5 was used (Goodman, 2001).
• Participant׳s mental health concurrent with the outcome at age 16 years, measured using the self-reported Short Moods and Feelings Questionnaire (SMFQ), administered in the same questionnaire as the self-harm questions. A cut off of 11 or more was taken as indicative of depressive symptoms (Patton et al., 2008).

Ethical approval to collect the outcome data and undertake the analysis was obtained from ALSPAC׳s Law and Ethics Committee, a registered Institutional Review Board.

### 2.3 Statistical analysis

#### 2.3.1 Multi-level analyses

Only 75% of respondents had data on school attended aged 14 years (collected from routine data sources for participants who had consented). One-way ANOVA models using this subsample suggested little clustering by school for the exposure measures (ICCs ranged from 0 to 0.026), or for the self-harm outcome (ICC=0.008). Therefore the main and secondary analyses were conducted using single level multivariable models so that the full sample could be used, but with the robust CIs reported throughout.

#### 2.3.2 Main analyses

The relationship between school risk factors aged 14 and self-harm aged 16 was assessed using multivariable logistic regression models. Each school-related variable was considered as the exposure in a logistic regression, adjusted for sex and SEP, with self-harm as the outcome. The remaining two confounders were added into each regression model incrementally, starting with the one expected to have the biggest effect. There was no statistical evidence that associations differed in males and females (*p*(interaction)>0.24 for all associations) and so all analyses combined data for males and females. To ensure that the outcome was current self-harm, those who reported self-harming but not in the past year were excluded from all analyses. A sensitivity analysis was done in which the main multivariable models were repeated including all those who had ever self-harmed.

The association between school-risk factors and mental health aged 16 was examined, and each fully adjusted model with self-harm as the outcome was repeated with participants׳ concurrent mental health included, to assess the extent to which poor mental health might explain the observed associations.

#### 2.3.3 Secondary analyses

Multinomial regression models were used to examine the effect of the exposures on self-harm with suicidal intent (defined as a positive response to ‘wanted to die’ the most recent time self-harmed and/or had ever seriously wanted to kill self when had self-harmed), and non-suicidal self-harm (NSSH), to see if school experiences were more strongly associated with one or other type of self-harming behaviour.

#### 2.3.4 Missing data imputation

**Fig. 1:**
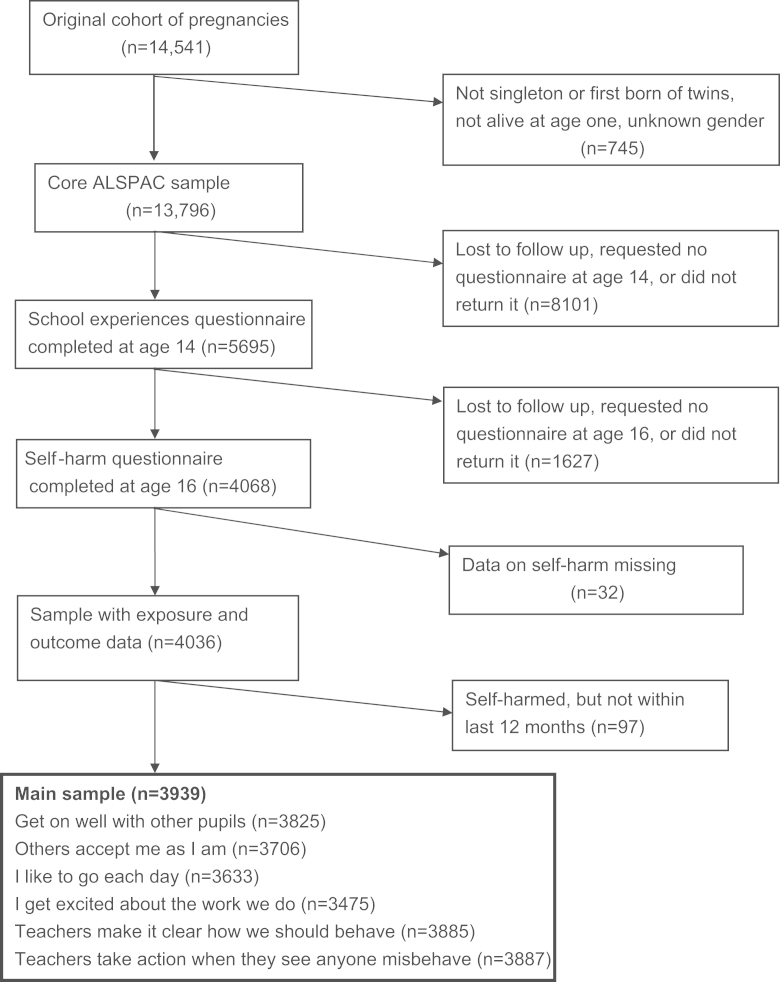
Flow of participants from pregnancy to the age 16 postal questionnaire in the ALSPAC cohort study.

We assessed the impact of non-response and missing data on our findings using Multivariate Imputation by Chained Equations (MICE) (Van Buuren et al., 1999) implemented using the ice routine (Royston, 2009) in Stata. This procedure creates multiple copies of the dataset and in each dataset replaces missing data with imputed values, sampled from their predictive distribution (Sterne et al., 2009). The validity of this approach assumes that data are Missing At Random (MAR), namely that conditional on the other data included in the imputation model, there should not be systematic differences between observed and missing values for a given variable. All variables used in the analyses (i.e. self-harm, school-related exposures and potential confounders) were included in the imputation models, along with a number of other variables: indicators of family adversity at enrolment such as home overcrowding and financial problems, earlier (more complete) measures of the confounders considered here, and measures of school experiences taken concurrently with the exposure variables and at two earlier time points (aged 11 years and 8 years). Missing data were imputed using linear or logistic regression models as appropriate. One hundred imputed datasets were derived, each entailing 20 cycles of regression switching. Imputation was conducted on the sample who had information on self-harm but incomplete data for the school-related exposure variables and other potential confounders (see Fig. 1).

## 3 Results

### 3.1 Respondents

In total, 5695 participants from the ALSPAC cohort completed a questionnaire about school related factors age 14, of whom 4068 (71%) returned the self-harm questionnaire at age 16. Among that 4068, 32 did not complete the questions regarding self-harm, leaving a sample of 4036 with both exposure and outcome data (see Fig. 1). The mean age of respondents at the time the exposure questionnaire was completed was 14 years and 3 months (standard deviation (SD) 2.7 months), and at the time the self-harm questions were completed was 16 years and 8 months (SD 2.9 months). Respondents were more likely than non-respondents from the original ALSPAC sample to be female, to have a mother in a non-manual social class, to live in a household with a high weekly income, and to have high educational qualifications compared to non-respondents (Kidger et al., 2012a).

Those who completed the self-harm questionnaire were more likely to agree that they liked to go to school at age 14 (*χ*^2^=33.44, *p*<0.001), that they were excited by the work that they do (*χ*^2^=12.54, *p*<0.001), that teachers make it clear how they should behave (*χ*^2^=7.51, *p*=0.006) and that teachers take action when they see someone misbehave (*χ*^2^=5.41, *p*=0.02), compared to those who had data at age 14 but did not complete the self-harm questionnaire.

Of the sample of 4036, 757 (18.8%) participants had ever self-harmed, and 660 (16.4%) had done so in the past year (see Fig. 1).

Table 1 shows the characteristics of those who had self-harmed in the past year by each of the exposure variables. Given the small numbers of participants selecting strongly disagree or strongly agree in some cases, all the exposure variables were re-categorised into binary variables (strongly agree or agree vs disagree or strongly disagree), with those who selected ‘don׳t know’ treated as missing.

### 3.2 Main results

There were no substantive differences between any of the results based on the imputed datasets, and those from the complete case analysis (see Appendices C and D), therefore results from the imputed datasets are reported. For each item measuring school experience, selecting “disagree” was associated with increased odds of self-harm in models controlling for sex, SEP, prior emotional health aged 13 and mother׳s mental health (Table 2, model 2). The strongest association was for the two connectedness items, followed by ‘teachers take action when they see anyone misbehave’. Adjusting for participant׳s emotional health aged 13 or mother׳s mental health had little impact on the strength of the association. Including those who had self-harmed but not in the previous year did not make any substantive difference to these associations (data not shown).

### 3.3 Adjusting for concurrent mental health

For all the exposure variables, poorer perceptions of one׳s school experience aged 14 were associated with increased odds of having a depression score on the SMFQ at age 16, once all confounders were controlled for (ORs ranged from 1.40 for ‘I get excited by the work that we do’ to 2.57 for ‘others accept me as I am’). When participants׳ mental health aged 16 was entered into the fully adjusted models, the association between school experiences and self-harm was somewhat attenuated (Table 2 model 3).

### 3.4 Self-harm with suicidal intent and NSSH

Of those who had self-harmed in the past year, 263 (39.8%) had experienced a desire to die or to kill themselves on at least one occasion (self-harm with suicidal intent). The associations between school experience and self-harm were similar for both self-harm with or without suicidal intent, but were generally stronger for self-harm with suicidal intent (Table 3). When NSSH was taken as baseline (Table 3 column 3), the odds of suicidal self-harm were greater for ‘get on well with other pupils’ (OR=1.73 [1.01, 2.96]), ‘teachers make it clear how we should behave’ (OR=1.68 [1.02, 2.79]), and ‘teachers take action when they see anyone misbehave’ (OR=1.53 [1.04, 2.26]).

## 4 Discussion

### 4.1 Main findings

Perceptions of the school environment at the age of 14 are associated with self-harm two years later. Specifically, individuals who do not feel connected to school (in terms of getting on well with and feeling accepted by others in their school), who do not enjoy going to school or the work done in class, and who do not perceive teachers to be consistent regarding rules and behaviour are more likely to self-harm in the future than those who do. The relationship between poor perceptions of one׳s school environment and self-harm is broadly the same for self-harm with or without suicidal intent, although there is some indication that negative experiences of school may be more strongly associated with suicidal self-harm.

### 4.2 Strengths and limitations

This is one of the few cohort studies to examine negative experiences of school as risk factors for adolescent self-harm, and the only one using data from England. As we did not have data on self-harm behaviour among this sample prior to age 14, it is not possible to be sure about the temporal order of events; it may be that the self-harm behaviour began first, leading to rejection by others at school for example, which then led to further self-harm However, it is known that self-harm rates rapidly increase throughout the teenage years, reaching a peak around the ages of 18/19 (Hawton et al., 2003), therefore the majority of self-harm episodes in this sample are likely to have occurred after the exposure data were collected, particularly as we excluded those who had self-harmed but not in the previous year. A second strength is the size of the dataset, which ensured good power to detect any effects, and its richness, which enabled a range of confounders to be controlled for, as well as several aspects of the school experience to be explored.

A limitation is that a large number of the original core cohort of 13,796 had been lost to follow up. This may have led to selection bias as non-respondents were more likely to be male, have a lower SEP, have performed less well in statutory exams aged 16, have poorer emotional health aged 13 and have a mother with a history of poor mental health (Kidger et al., 2012a). Further, those who completed the questions on self-harm were more likely to enjoy school and perceive teachers to be fair, compared to those who did not complete these questions, although there was no difference between these two groups on how connected they felt to school. A second limitation is that some aspects of the school environment likely to be important for future self-harming behaviour – notably experiences of bullying (Winsper et al., 2012) – were not measured in the 14 years questionnaire, and therefore could not be examined. Relatedly, given that the confounder measures were self-report, there may be some measurement error leading to residual confounding. A final shortcoming is the fact that the exposure and outcome data were also self-report. It is not uncommon for self-harm to be examined in this way, indeed it has been suggested that this leads to greater accuracy than interviews, where respondents may underreport this behaviour (Safer, 1997). However, low mood may be associated both with increased retrospective reporting of self-harm and a more negative perception of school experience, thereby exaggerating the relationship between the two. Further, by measuring school factors via self-report the study was only able to examine participants׳ *perceptions* of school as a predictor of self-harm; unpicking any separate effect of the school environment as defined by a more objective measure was beyond its scope.

### 4.3 Relevance to the wider literature

The findings strengthen previous cross-sectional evidence that relationships within schools are associated with self-harm (Landstedt and Gillander Gadin, 2011; McMahon et al., 2010; Winfree and Jiang, 2010; Wichstrom, 2009). A smaller longitudinal study in Scotland found poor connectedness – in the form of lack of involvement and engagement with school – predicted increased odds of self-harm (Young et al., 2011). Our study also highlights the potential importance of other aspects of the school experience that have been less well explored. One cross sectional study found that a perception of teachers as fair was protective for self-harm (Carter et al., 2007), and others have found that perceptions of school as hostile, which may be related to the two “fair environment” items here, are associated with an increased risk of self-harm (Landstedt and Gillander Gadin, 2011; Winfree and Jiang, 2010). The importance of clear boundaries and happiness at school for self-harm have not previously been examined, but both have been found to predict lower depression (Way et al., 2007, Van Voorhees et al., 2008).

The small amount of clustering by school indicates that what has been measured in this study is subjective perceptions of the school experience, and that those individual perceptions are not necessarily an accurate representation of the actual school environment. School-level effects have been found to explain only a small amount of the variance of self-harm and other health outcomes once individual effects are controlled for (Hankin and Abela, 2011; Young et al., 2011; Saab and Klinger 2010; Bond et al., 2004). This suggests that future interventions seeking to reduce the risk of self-harm in the school context must focus on changing the ways in which vulnerable individuals experience and interact with their school environment, rather than merely changing the environment.

The partial attenuation of the association between school experience and self-harm when current mental health is adjusted for may be explained by an underlying pathology of which depressed mood and self-harm are both outcomes. Or it may be that poor school experience leads to depressed mood, which in turn leads individuals to self-harm; regulation of difficult feelings has been reported as the most frequently endorsed motivation for self-harm (Kidger et al., 2012a; Laye-Gindhu and Schonert-Reichl, 2005). However, a degree of association remained for most of the exposures even when mental health was adjusted for. This may be explained in part by some of the mediating effects of depression being missed if depressive symptoms developed following difficult school experiences and led to self-harm, but then subsided by the time the SMFQ was administered two years later. But it may also indicate that difficult experiences at school lead to feelings such as anxiety or anger, that are sufficiently intense and distressing to lead to self-harm (Young et al., 2007), but that would not necessarily be evident in the SMFQ, which only measures depressive symptoms. The exact nature and direction of these complex relationships between school experience, mental health and self-harm could only be further clarified with a more detailed pathways analysis, in which measures of all three plus confounders are included at several time points.

A growing number of studies have distinguished between NSSH and suicidal behaviour, producing evidence of differences between the two in terms of risk factors and the functions served (Wichstrom, 2009; Mars et al., 2014). The findings from this study suggest that NSSH and suicidal behaviour generally share the same risk factors in relation to school experiences, although this relationship may be stronger for suicidal self-harm.

### 4.4 Implications

Given the independent relationship between poor school experiences and self-harm, assessing students׳ perceptions of connectedness, enjoyment and fairness at school may provide a method for identifying those who are at risk of self-harm, and who may particularly benefit from school-based interventions designed to promote mental health and support healthy coping strategies. Further examination of the relationship between school experiences and later self-harm is needed to clarify the extent to which the former causes the latter, or whether they are both outcomes of the same underlying distress. An important development to assist with this understanding would be a validated scale that brings together all the aspects of school experience that have been associated with self-harm in previous studies. If poor school experience is a contributory factor for self-harm, then interventions that improve students׳ sense of connectedness, enjoyment and fairness at school may reduce their risk of self-harm at a later date.

## Conflict of interest

None of the authors declare any conflict of interest.

## Role of funding source

The UK Medical Research Council and Wellcome Trust (Grant ref. 092731) and the University of Bristol provide core support for the ALSPAC study. This particular analysis was funded by a Medical Research Council postdoctoral fellowship awarded to Judi Kidger. The funding bodies had no involvement in the study design, the collection, analysis and interpretation of data, the writing of the report or the decision to submit the article for publication.

## Figures and Tables

**Table 1:** Proportion of sample who have self-harmed (SH) in the last year, by risk factors aged 14 years. Exposure variables all begin with ‘My school is a place where…’.

**Factor**	**Items**	**SH aged 16**, ***N*****=660**		**No SH aged 16**, ***N*****=3279**		**Total**^a^, ***N*****=3939**	
		***N***	**%**	***N***	**%**	***N***	**%**
*Connectedness to school and other students*	*I get on well with other pupils in my class*						
	Strongly agree	178	*27.7*	1084	*34.1*	1262	*33.0*
	Agree	406	*63.2*	1972	*62.0*	2378	*62.2*
	Disagree	47	*7.3*	106	*3.3*	153	*4.0*
	Strongly disagree	11	*1.7*	21	*0.7*	32	*0.8*
	*Others accept me as I am*						
	Strongly agree	99	*15.9*	747	*24.2*	846	*22.8*
	Agree	370	*59.3*	2034	*66.0*	2404	*64.9*
	Disagree	115	*18.4*	252	*8.2*	367	*9.9*
	Strongly disagree	40	*6.4*	49	*1.6*	89	*2.4*
*Enjoyment of school*	*I like to go each day*						
	Strongly agree	32	*5.3*	257	*8.5*	289	*8.0*
	Agree	340	*55.8*	1834	*60.7*	2174	*59.8*
	Disagree	187	*30.7*	801	*26.5*	988	*27.2*
	Strongly disagree	50	*8.2*	132	*4.4*	182	*5.0*
	*I get excited about the work we do*						
	Strongly agree	17	*2.9*	77	*2.7*	94	*2.7*
	Agree	140	*23.5*	886	*30.8*	1026	*29.5*
	Disagree	350	*58.7*	1621	*56.3*	1971	*56.7*
	Strongly disagree	89	*14.9*	295	*10.3*	384	*11.1*
*Clear/fair boundaries*	*Most teachers make it clear how we should behave*						
	Strongly agree	138	*21.2*	873	*27.0*	1011	*26.0*
	Agree	445	*68.3*	2134	*66.0*	2579	*66.4*
	Disagree	62	*9.5*	212	*6.6*	274	*7.1*
	Strongly disagree	7	*1.1*	14	*0.4*	21	*0.5*
	*Most teachers take action when they see anyone misbehave*						
	Strongly agree	118	*18.1*	778	*24.1*	896	*23.1*
	Agree	402	*61.6*	2099	*64.9*	2501	*64.3*
	Disagree	121	*18.5*	319	*9.9*	440	*11.3*
	Strongly disagree	12	*1.8*	38	*1.2*	50	*1.3*

a Excluding those who had missing data or selected “don’t know” in response to the exposure question (range from 53 for teachers take action to 468 for get excited about the work we do).

**Table 2:** Multivariable models showing the odds ratios for self-harm aged 16 for each exposure variable at age 14^a^.

	**Imputed data analysis (*****N*****=4742)**		
	**Model 1**	**Model 2**	**Model 3**

*Get on well with other pupils*			
Agree	1.00	1.00	1.00
Disagree	2.53 (1.83, 3.48)	2.43 (1.76, 3.35)	1.97 (1.39, 2.80)
*p value*	*<0.001*	*<0.001*	*<0.001*
*Others accept me as I am*			
Agree	1.00	1.00	1.00
Disagree	2.80 (2.25, 3.48)	2.69 (2.16, 3.35)	2.18 (1.72, 2.76)
*p value*	*<0.001*	*<0.001*	*<0.001*
*A place I like to go*			
Agree	1.00	1.00	1.00
Disagree	1.43 (1.19, 1.72)	1.40 (1.17, 1.69)	1.23 (1.01, 1.49)
*p value*	*<0.001*	*<0.001*	*0.037*
*Excited about the work we do*			
Agree	1.00	1.00	1.00
Disagree	1.38 (1.12, 1.69)	1.36 (1.10, 1.67)	1.22 (0.98, 1.51)
*p value*	*0.002*	*0.004*	*0.76*
*Clear how we should behave*			
Agree	1.00	1.00	1.00
Disagree	1.62 (1.22, 2.15)	1.59 (1.20, 2.12)	1.36 (1.00, 1.84)
*p value*	*0.001*	*0.001*	*0.049*
*Teachers take action*			
Agree	1.00	1.00	1.00
Disagree	2.53 (1.83, 3.48)	1.89 (1.51, 2.37)	1.60 (1.26, 2.04)
*p value*	*<0.001*	*<0.001*	*p<0.001*

Model 1: adjusted for sex and SEP. Model 2: adjusted for sex, SEP, own emotional health aged 13, and mother׳s mental health aged 11. Model 3: adjusted for sex, SEP, own emotional health aged 13, mother׳s mental health aged 11 and own mental health aged 16.

a Participants who had self-harmed but not in the last year excluded in all models.

**Table 3:** Multinomial models examining association between school risk factors aged 14 and (a) each type of self-harm compared to no self-harm, and (b) self-harm with suicidal intent compared to non-suicidal self-harm (NSSH)a,b.

**Imputed data analysis (*****N*****=4742)**				
**Variables**	**No self-harm as baseline**		**NSSH as baseline**	***P*****value from omnibus test**
	**SH with suicidal intent**	**NSSH**	**SH with suicidal intent**	
*Get on well with other pupils*				
Agree	1.00	1.00	1.00	<0.0001
Disagree	3.26 (2.15, 4.94)	1.88 (1.23, 2.89)	1.73 (1.01, 2.96)	
*Others accept me as I am*				
Agree	1.00	1.00	1.00	<0.0001
Disagree	3.00 (2.19, 4.11)	2.50 (1.92, 3.25)	1.20 (0.82, 1.74)	
*A place I like to go*				
Agree	1.00	1.00	1.00	0.0007
Disagree	1.57 (1.20, 2.06)	1.30 (1.04, 1.64)	1.21 (0.87, 1.68)	
*Excited by the work that we do*				
Agree	1.00	1.00	1.00	0.0143
Disagree	1.34 (0.98, 1.84)	1.37 (1.06, 1.76)	0.98 (0.67, 1.44)	
*Clear how we should behave*				
Agree	1.00	1.00	1.00	0.0006
Disagree	2.13 (1.45, 3.13)	1.26 (0.87, 1.84)	1.68 (1.02, 2.79)	
*Teachers take action*				
Agree	1.00	1.00	1.00	<0.0001
Disagree	2.42 (1.78, 3.29)	1.58 (1.18, 2.11)	1.53 (1.04, 2.26)	

a One participant did not complete suicidal intent questions and was omitted from the analysis.

b Models adjusted for sex, SEP, own emotional health aged 13 and mother׳s mental health aged 11.

## References

[bib101] Bond L., Patton G., Glover S., Carlin J.B., Butler H., Thomas L., Bowes G. (2004). The Gatehouse Project: can a multilevel school intervention affect emotional wellbeing and health risk behaviours?. J. Epidemiol. Community Health.

[bib2] Boyd A., Golding J., Macleod J., Lawlor D.A., Fraser A., Henderson J., Molloy L., Ness A., Ring S., Davey Smith G. (2013). Cohort profile: the ׳Children of the 90s׳ – the index offspring of the Avon longitudinal study of parents and children. Int. J. Epidemiol..

[bib3] Carter M., McGee R., Taylor B., Williams S. (2007). Health outcomes in adolescence: associations with family, friends and school engagement. J. Adolesc..

[bib5] Cox J.L., Chapman G., Murray D., Jones P. (1996). Validation of the Edinburgh Postnatal Depression Scale (EPDS) in nonpostnatal women. J. Affect. Disord..

[bib6] Cox J.L., Holden J.M., Sagovsky R. (1987). Detection of postnatal depression: development of the 10-item Edinburgh Postnatal Depression Scale. Br. J. Psychiatry.

[bib7] Denny S.J., Robinson E.M., Utter J., Fleming T.M., Grant S., Milfont T.L., Crengle S., Ameratunga S.N., Clark T. (2011). Do schools influence student risk-taking behaviors and emotional health symptoms?. J. Adolesc. Health.

[bib8] Fleming T.M., Merry S.N., Robinson E.M., Denny S.J., Watson P.D. (2007). Self-reported suicide attempts and associated risk and protective factors among secondary school students in New Zealand. Aust. N. Z. J. Psychiatry.

[bib9] Fliege H., Lee J.R., Grimm A., Klapp B.F. (2009). Risk factors and correlates of deliberate self-harm behaviour: a systematic review. J. Psychosom. Res..

[bib10] Foley D.L., Goldston D.B., Costello J., Angold A. (2006). Proximal psychiatric risk factors for suicidality in youth: the Great Smoky Mountains Study. Arch. Gen. Psychiatry.

[bib11] Fraser A., Macdonald-Wallis C., Tilling K., Boyd A., Golding J., Davey Smith G., Henderson J., Macleod J., Molloy L., Ness A., Ring S., Nelson S.M., Lawlor D.A. (2013). Cohort profile: the Avon longitudinal study of parents and children. ALSPAC Mothers Cohort. Int. J. Epidemiol..

[bib12] Goodman R. (2001). Psychometric properties of the strengths and difficulties questionnaire. J. Am. Acad. Child Adolesc. Psychiatry.

[bib13] Hankin B.L., Abela J.R.Z. (2011). Nonsuicidal self-injury in adolescence: prospective rates and risk factors in a 2½ year longitudinal study. Psychiatry Res..

[bib15] Hawton K., Hall S., Simkin S., Bale E., Bond A., Codd S., Steward A. (2003). Deliberate self-harm in adolescents: a study of characteristics and trends in Oxford, 1990–2000. J. Child Psychol. Psychiatry.

[bib16] Hawton K., Zahl D. (2003). Suicide following deliberate self-harm: long-term follow-up of patients who presented to a general hospital. Br. J. Psychiatry.

[bib17] Hawton K., Rodham K., Evans E., Weatherall R. (2002). Deliberate self-harm in adolescents: self-report survey in schools in England.

[bib18] Kidger J., Heron J., Lewis G., Evans J., Gunnell D. (2012). Adolescent self-harm and suicidal thoughts in the ALSPAC cohort: a self-report survey in England. BMC Psychiatry.

[bib19] Kidger J., Araya R., Donovan J., Gunnell D. (2012). The effect of the school environment on the emotional health of adolescents: a systematic review. Pediatrics.

[bib20] Landstedt E., Gillander Gadin K. (2011). Deliberate self-harm and associated factors in 17-year-old Swedish students. Scand. J. Public Health.

[bib21] Laye-Gindhu A., Schonert-Reichl K.A. (2005). Nonsuicidal self-harm among community adolescents: understanding the “whats” and “whys” of self-harm. J. Youth Adolesc..

[bib22] Mars B., Heron J., Crane C., Hawton K., Kidger J., Lewis G., Macleod J., Tilling K., Gunnell D. (2014). Differences in risk factors for self-harm with and without suicidal intent: findings from the ALSPAC cohort. J. Affect. Disord..

[bib23] McMahon E.M., Reulbach U., Corcoran P., Keeley H.S., Perry I.J., Arensman E. (2010). Factors associated with deliberate self-harm among Irish adolescents. Psychol. Med..

[bib24] Patton G.C., Olsson C., Bond L., Toumbourou J.W., Carlin J.B., Hemphill S.A., Catalano R.F. (2008). Predicting female depression across puberty: a two-nation longitudinal study. J. Am. Acad. Child Adolesc. Psychiatry.

[bib25] Patton G.C., Bond L., Carlin J.B., Thomas L., Butler H., Glover S., Catalano R., Bowes G. (2006). Promoting social inclusion in schools: a group-randomized trial of effects on student health risk behavior and well-being. Am. J. Public Health.

[bib26] Royston P. (2009). Multiple imputation of missing values: further update of ice, with an emphasis on categorical variables. Stata J..

[bib27] Saab H., Klinger D. (2010). School differences in adolescent health and wellbeing: findings from the Canadian health behaviour in school-aged children study. Soc. Sci. Med..

[bib539] Safer D.J. (1997). Self-reported suicide attempts by adolescents. Ann. Clinl. Psychiatry.

[bib28] Shochet I.M., Dadds M.R., Ham D., Montague R. (2006). School connectedness is an underemphasized parameter in adolescent mental health: results of a community prediction study. J. Clin. Child Adolesc. Psychol..

[bib29] Sterne J.A.C., White I.R., Carlin J.K., Spratt M., Royston P., Kenward M.G., Wood A.M., Carpente r.J.R. (2009). Multiple imputation for missing data in epidemiological and clinical research: potential and pitfalls. Br. Med. J..

[bib30] Van Buuren S., Boshuizen H.C., Knook D.L. (1999). Multiple imputation of missing blood pressure covariates in survival analysis. Stat. Med..

[bib31] Van Voorhees B.W., Paunesku D., Kuwabara S.A., Basu A., Gollan J., Hankin B.L., Melkonian S., Reinecke M. (2008). Protective and vulnerability factors predicting new-onset depressive episode in a representative of US adolescents. J. Adolesc. Health.

[bib32] Wang M.T. (2009). School climate support for behavioral and psychological adjustment: testing the mediating effect of social competence. Sch. Psychol. Q..

[bib33] Way N., Reddy R., Rhodes J. (2007). Students׳ perceptions of school climate during the middle school years: associations with trajectories of psychological and behavioral adjustment. Am. J. Community Psychol..

[bib34] Wichstrom L. (2009). Predictors of non-suicidal self-injury versus attempted suicide: similar or different?. Arch. Suicide Res..

[bib35] Winfree L., Jiang S. (2010). Youthful suicide and social support. Youth Viol. Juv. Justice.

[bib542] Winsper C., Lereya T., Zanarini M., Wolke D. (2012). Involvement in bullying and suicide-related behavior at 11 years: a prospective birth cohort study. J. Am. Acad. Child Adolesc. Psychiatry.

[bib36] Young R., Sweeting H., Ellaway A. (2011). Do schools differ in suicide risk? The influence of school and neighbourhood on attempted suicide, suicidal ideation and self-harm among secondary school pupils. BMC Public Health.

[bib37] Young R., Van Beinum M., Sweeting H., West P. (2007). Young people who self-harm. Br. J. Psychiatry.

