# Supplementary material for: Self-reported school experience as a predictor of self-harm during adolescence: A prospective cohort study in the South West of England (ALSPAC)
Source: J Affect Disord. 2015 Mar 1;173:163–9. doi: 10.1016/j.jad.2014.11.003 (PMC4286629; doi:10.1016/j.jad.2014.11.003)
Supplement: Supplementary file 1 — Supplementary data [file mmc1.pdf]

APPENDIX A: School related questions in 14 year questionnaire

Section A: About you and your school

A1. These questions ask about how you feel about going to school and what it's like being there:

My school is a place where…	Strongly Agree	Agree	Disagree	Strongly Disagree	Don't Know
↓					
**a) I really like to go each day**					
**b) I learn to get along with other people**					
**c) Other pupils accept me as I am**					
**d) I like to be**					
**e) I like to do extra work**					
**f) I feel happy**					
**g) I feel lonely**					
**h) I feel proud to be a pupil**					
**i) I feel worried**					
**j) People trust me**					
**k) I have a lot of fun**					
**l) I enjoy what I do in class**					
**m) I can learn what I need to know**					
**n) I get excited about the work we do**					
**o) I get upset**					
**p) I know people think a lot of me**					
**q) I get on well with other pupils in my classes**					
**r) People can depend on me**					
**s) Other pupils are very friendly**					
**t) I feel restless**					

A2 Again please tell us how much you agree or disagree with each of the following statements:

	Strongly Agree	Agree	Disagree	Strongly Disagree
**a) Most teachers in my school take action when they see anyone breaking the school rules**				
**b) Most teachers at my school make it clear how we should behave**				
**c) Most of my teachers don't really listen to what I say in class**				
**d) In this school, most teachers and pupils really trust one another**				
**e) I get treated unfairly by most of my teachers**				
**f) Most of my teachers make sure we do any homework that is set**				
**g) Most of my teachers can keep order in class**				
**h) People think my school is a good school**				
**i) The work I do in lessons is interesting to me**				
**j) I like most of my teachers**				
**k) Most of my teachers treat everyone the same regardless of skin colour or cultural background**				
**l) Most of my teachers praise me when I do my school work well**				
**m) Most teachers at my school treat pupils with respect**				
**n) Most teachers at my school have given up on some of the pupils**				
**o) Most teachers at my school believe that all pupils can learn**				
**p) If I get caught breaking school rules, I'm more likely to be punished than others**				
**q) Compared with other pupils in my classes, most of my teachers are more likely to take an interest in my work**				

A3. Next, please think about your school work:

	Very good	Above average	Average	Below average	Not at all good	Don't know
**a) How would you describe your schoolwork?**						
**b) How do you think your teachers would describe your schoolwork?**						

APPENDIX B

Factor analysis of the questionnaire items: only items with a rotated component loading of 0.6 or more are shown for each factor

RESPONDENT'S SCHOOL IS A PLACE WHERE…	CONNECTEDNESS TO SCHOOL/STUDENTS	ENJOYMENT OF SCHOOL	FAIR/CLEAR BOUNDARIES
they get on well with other pupils in their class	0.861		
other pupils accept them	0.752		
other pupils are very friendly	0.734		
people can depend on them	0.724		
people trust them	0.688		
they know people who think a lot of me	0.665		
they have a lot of fun	0.606		
they really like to go each day		0.721	
they get excited about the work they do		0.702	
they enjoy what they do in class		0.690	
teachers make it clear how they should behave			0.874
teachers take action when they see anyone breaking school rules			0.835
teachers keep the class in order			0.721
teachers at school treats pupils with respect			0.718
teachers at school believe that all pupils can learn			0.680
respondent makes sure that any homework set is completed			0.633
teachers praise them when they do school work well			0.628

Appendix C

Multivariable models examining association between school risk factors aged 14 and self-harm aged 16,[1] comparison of complete case analysis and imputed data analysis.

	**Complete case analysis[2]**			**Imputed data analysis (N=4742)**		
	**Model 1**	**Model 2**	**Model 3**	**Model 1**	**Model 2**	**Model 3**
Get on well with other pupils						
	N=2476		N=2441			
Agree	1.00	1.00	1.00	1.00	1.00	1.00
Disagree	2.12 (1.39, 3.24)	2.15 (1.38, 3.36)	1.67 (1.00, 2.79)	2.53 (1.83, 3.48)	2.43 (1.76, 3.35)	1.97 (1.39, 2.80)
p value	*0.001*	*0.001*	*0.05*	*<0.001*	*<0.001*	*<0.001*
Others accept me as I am						
	N=2401		N=2365			
Agree	1.00	1.00	1.00	1.00	1.00	1.00
Disagree	2.61 (1.95, 3.50)	2.58 (1.93, 3.44)	2.05 (1.50, 2.82)	2.80 (2.25, 3.48)	2.69 (2.16, 3.35)	2.18 (1.72, 2.76)
p value	*<0.0001*	*<0.001*	*<0.001*	*<0.001*	*<0.001*	*<0.001*
A place I like to go N=2355						
	N=2355		N=2322			
Agree	1.00	1.00	1.00	1.00	1.00	1.00
Disagree	1.45 (1.15, 1.81)	1.50 (1.18, 1.90)	1.31 (1.02, 1.69)	1.43 (1.19, 1.72)	1.40 (1.17, 1.69)	1.23 (1.01, 1.49)
p value	*0.001*	*0.001*	*0.038*	*<0.001*	*<0.001*	*0.037*
Excited about the work we do						
	N=2257		N=2227			
Agree	1.00	1.00	1.00	1.00	1.00	1.00
Disagree	1.25 (0.98, 1.59)	1.22 (0.95, 1.57)	1.10 (0.85, 1.43)	1.38 (1.12, 1.69)	1.36 (1.10, 1.67)	1.22 (0.98, 1.51)
p value	*0.069*	*0.115*	*0.479*	*0.002*	*0.004*	*0.76*
Clear how we should behave						
	N=2513		N=2475			
Agree	1.00	1.00	1.00	1.00	1.00	1.00
Disagree	1.21 (0.82, 1.79)	1.21 (0.80, 1.83)	1.01 (0.65, 1.57)	1.62 (1.22, 2.15)	1.59 (1.20, 2.12)	1.36 (1.00, 1.84)
p value	*0.344*	*0.357*	*0.954*	*0.001*	*0.001*	*0.049*
Teachers take action						
	N=2515		N=2477			
Agree	1.00	1.00	1.00	1.00	1.00	1.00
Disagree	2.07 (1.56, 2.75)	1.91 (1.43, 2.56)	1.58 (1.16, 2.15)	2.53 (1.83, 3.48)	1.89 (1.51, 2.37)	1.60 (1.26, 2.04)
p value	*<0.001*	*<0.001*	*0.004*	*<0.001*	*<0.001*	*p<0.001*

[1]Excluding participants who had self-harmed but not in the last year in all models
[2]Only participants with complete data for all variables included
Model 1: adjusted for sex and SEP
Model 2: adjusted for sex, SEP, own emotional health aged 13, and mother's mental health aged 11
Model 3: adjusted for sex, SEP, own emotional health aged 13, mother's mental health aged 11 and own mental health aged 16

Appendix D

Table 3: Multinomial models examining association between school risk factors aged 14 and a) each type of self-harm compared to no self-harm b) self-harm with suicidal intent compared to self-harm without suicidal intent (NSSI)[1 2]

	Complete case analysis				Imputed data analysis (N=4742)			
	**No self-harm as baseline**		**NSSI as baseline**	**P value from omnibus test**	**No self-harm as baseline**		**NSSI as baseline**	**P value from omnibus test**
**Variables**	**Suicidal intent**	**NSSI**	**Suicidal intent**		**Suicidal intent**	**NSSI**	**Suicidal intent**	
Get on well with other pupils N=2475								
Agree	1.00	1.00	1.00	0.0019	1.00	1.00	1.00	<0.0001
Disagree	2.61 (1.44, 4.71)	1.87 (1.06, 3.28)	1.40 (0.67, 2.90)		3.26 (2.15, 4.94)	1.88 (1.23, 2.89)	1.73 (1.01, 2.96)	
Others accept me as I am N=2400								
Agree	1.00	1.00	1.00	<0.0001	1.00	1.00	1.00	<0.0001
Disagree	2.45 (1.60, 3.77)	2.66 (1.90, 3.74)	0.92 (0.56, 1.51)		3.00 (2.19, 4.11)	2.50 (1.92, 3.25)	1.20 (0.82, 1.74)	
A place I like to go N=2354								
Agree	1.00	1.00	1.00	0.0033	1.00	1.00	1.00	0.0007
Disagree	1.43 (0.99, 2.08)	1.54 (1.17, 2.04)	0.93 (0.60, 1.44)		1.57 (1.20, 2.06)	1.30 (1.04, 1.64)	1.21 (0.87, 1.68)	
Excited by the work that we do N=2257								
Agree	1.00	1.00	1.00	0.2189	1.00	1.00	1.00	0.0143
Disagree	1.08 (0.74, 1.58)	1.31 (0.97, 1.78)	0.82 (0.52, 1.30)		0.98 (0.67, 1.44)	1.37 (1.06, 1.76)	0.98 (0.67, 1.44)	
Clear how we should behave N=2512								
Agree	1.00	1.00	1.00	0.0252	1.00	1.00	1.00	0.0006
Disagree	1.95 (1.16, 3.29)	0.80 (0.45, 1.43)	2.43 (1.17, 5.02)		2.13 (1.45, 3.13)	1.26 (0.87, 1.84)	1.68 (1.02, 2.79)	
Teachers take action N=2514								
Agree	1.00	1.00	1.00	<0.0001	1.00	1.00	1.00	<0.0001
Disagree	2.36 (1.56, 3.57)	1.68 (1.14, 2.38)	1.41 (0.85, 2.33)		2.42 (1.78, 3.29)	1.58 (1.18, 2.11)	1.53 (1.04, 2.26)	

[1]One participant left both questions blank concerning suicidal intent and was omitted from the analysis
[2]Models adjusted for sex, SEP, own emotional health aged 13 and mother's mental health aged 11
